# Effects of social defeat stress on dopamine D2 receptor isoforms and proteins involved in intracellular trafficking

**DOI:** 10.1186/s12993-018-0148-5

**Published:** 2018-10-08

**Authors:** Vishwanath Vasudev Prabhu, Thong Ba Nguyen, Yin Cui, Young-Eun Oh, Keon-Hak Lee, Tarique R. Bagalkot, Young-Chul Chung

**Affiliations:** 1 Department of Psychiatry, Chonbuk National University Medical School, Jeonju, 561-756 South Korea; 2 Research Institute of Clinical Medicine of Chonbuk National University-Biomedical Research Institute of Chonbuk National University Hospital, Jeonju, 561-756 South Korea; 3 Department of Psychiatry, Maeumsarang Hospital, 465-23, Wanju, Jeollabuk-do South Korea; 4 Shanghai Mental Health Center, Shanghai Jiao Tong University of Medicine, 600 Wan Ping Nan Road, Shanghai, 200013 P. R. China; 5 Department of Cell Biology, University of Pittsburgh, 200 Lothrop Street, Biomedical Science Tower S372, Pittsburgh, PA 15213 USA

**Keywords:** Dopamine receptor isoforms, Elevated plus maze, GASP-1, Rab4, Social behaviors, Social defeat stress

## Abstract

**Background:**

Chronic social defeat stress induces depression and anxiety-like behaviors in rodents and also responsible for differentiating defeated animals into stress susceptible and resilient groups. The present study investigated the effects of social defeat stress on a variety of behavioral parameters like social behavior, spatial learning and memory and anxiety like behaviors. Additionally, the levels of various dopaminergic markers, including the long and short form of the D2 receptor, and total and phosphorylated dopamine and cyclic adenosine 3′,5′-monophosphate regulated phosphoprotein-32, and proteins involved in intracellular trafficking were assessed in several key brain regions in young adult mice.

**Methods:**

Mouse model of chronic social defeat was established by resident-intruder paradigm, and to evaluate the effect of chronic social defeat, mice were subjected to behavioral tests like spontaneous locomotor activity, elevated plus maze (EPM), social interaction and Morris water maze tests.

**Results:**

Mice were divided into susceptible and unsusceptible groups after 10 days of social defeat stress. The susceptible group exhibited greater decreases in time spent in the open and closed arms compared to the control group on the EPM. In the social interaction test, the susceptible group showed greater increases in submissive and neutral behaviors and greater decreases in social behaviors relative to baseline compared to the control group. Furthermore, increased expression of D2L, D2S, Rab4, and G protein-coupled receptor associated sorting protein-1 was observed in the amygdala of the susceptible group compared to the control group.

**Conclusion:**

These findings suggest that social defeat stress induce anxiety-like and altered social interacting behaviors, and changes in dopaminergic markers and intracellular trafficking-related proteins.

**Electronic supplementary material:**

The online version of this article (10.1186/s12993-018-0148-5) contains supplementary material, which is available to authorized users.

## Background

Social defeat is the result of a confrontation between male animals and is an ethologically relevant experimental paradigm that can be used to understand the physiological and behavioral adaptations to repeated social stress. The social defeat stress paradigm has been widely used as an animal model for depression, anxiety disorders, and drug abuse [1, 2]. This paradigm may also be useful for identifying the environmental factors associated with schizophrenia given that social defeat results in deficits in prepulse inhibition [3], an enhanced mesocorticolimbic dopamine response [4, 5], increased phasic activity in ventral tegmental area (VTA) dopaminergic neurons [6], reductions in striatal dopamine transporter (DAT) binding [7], and behavioral and neuronal cross-sensitization to amphetamine [8].

Social defeat stress also induces depression-like behaviors, such as a reduced sucrose preference, decreased social interaction and, and enhances anxiety-like behaviors [9, 10], such as more time spent in a dark box in the light/dark preference test [11] and an enhanced and prolonged response in the acoustic startle test [12]. For the present study, a particular focus was placed on changes in social behaviors, including dominant and submissive behaviors, and social avoidance because these symptoms are relatively commonly observed in patients with depressive disorder or schizophrenia. Several studies have addressed this issue but most have used rats [13] rather than mice [6, 14].

Two isoforms of the dopamine D2 receptor (D2R) have been identified; a long form (D2L) and a short form (D2S; [15]). The two isoforms are generated by alternative splicing of the same gene but show differential distributions [16] and functions [17, 18]. In the postmortem brains of patients with schizophrenia, there are changes in the mRNA levels of both D2S and D2L: increased expression of D2S in the dorsolateral prefrontal cortex (DLPFC) [19] and mixed results on D2L in the DLPFC and frontal cortex [19, 20]. However, to date, no studies have investigated the effects of social defeat stress on D2L or D2S except for one study from our research group [21], which found increased expression of D2S and D2L in the prefrontal cortex (PFC) of susceptible mice compared to controls. Dopamine and cyclic adenosine 3′,5′-monophosphate-regulated phosphoprotein-32 (DARPP-32) play central roles in mediating the effects of dopamine and glutamate [22, 23] and their expression can be altered by acute stress [24] and electroconvulsive stimulation [25]. Our research group reported significant increases in the expression of total DARPP-32 and phosphorylated DARPP-32 (p-DARPP-32) in the PFC and amygdala (AMY) of defeated mice [26]. However, those studies did not separate defeated mice into susceptible and unsusceptible groups, which is important for exploring the mechanisms that underlie the susceptibility to stress.

Schubert et al. [27] proposed that abnormalities in clathrin-mediated endocytosis and protein trafficking are core pathophysiological processes associated with schizophrenia and bipolar disorder. Of the various proteins involved in the trafficking of dopamine receptors, three are of particular interest: ADP-ribosylation factor 6 (ARF-6; [28]), Rab proteins [29] and G protein-coupled receptor (GPCR) associated sorting protein-1 (GASP-1; [30]) because they are involved in the regulation of vesicular traffic and organelle structure and associated with the degradation of D2Rs respectively.

Thus, the present study aimed to investigate the effects of chronic social defeat on a variety of behavioral parameters, including the social interaction, EPM, and MWM tests, and the levels of dopaminergic markers (D2L, D2S, and total and p-DARPP-32) and proteins involved in intracellular trafficking in several brain regions of mice known to be affected in stress-related disorders such as anxiety and depressive disorders [31], and schizophrenia [32].

## Methods

### Animals

The social defeat procedure included male C57BL/6N mice and male CD1 (ICR) mice (Orient Company; Seongnam, South Korea) aged 6 and 15 weeks, respectively, and weighing 18–22 and 40–44 g, respectively, at the time of arrival. The C57BL/6N mice were group-housed while the CD1 mice were single-housed. The social interaction test included CD1 mice (4 weeks old) with similar weights that were matched to the C57BL/6N mice. All animals were housed in temperature-controlled rooms at 22 °C under a 12 h light/dark cycle with ad libitum food and water and were handled daily for 1 week to minimize stress during the behavioral experiments.

All the protocols in this experiment complied with the National Institutes of Health’s Guide for the Care and Use of Laboratory Animals (NIH Pub. No. 85-23, revised 1996) [33]. The entire project was reviewed and approved by the Institutional Animal Care and Use Committee (cuh-IACUC-151027-32) of Chonbuk National University Medical School on the basis of 3Rs (replacement, refinement and reduction).

### Study design

**Fig. 1:**
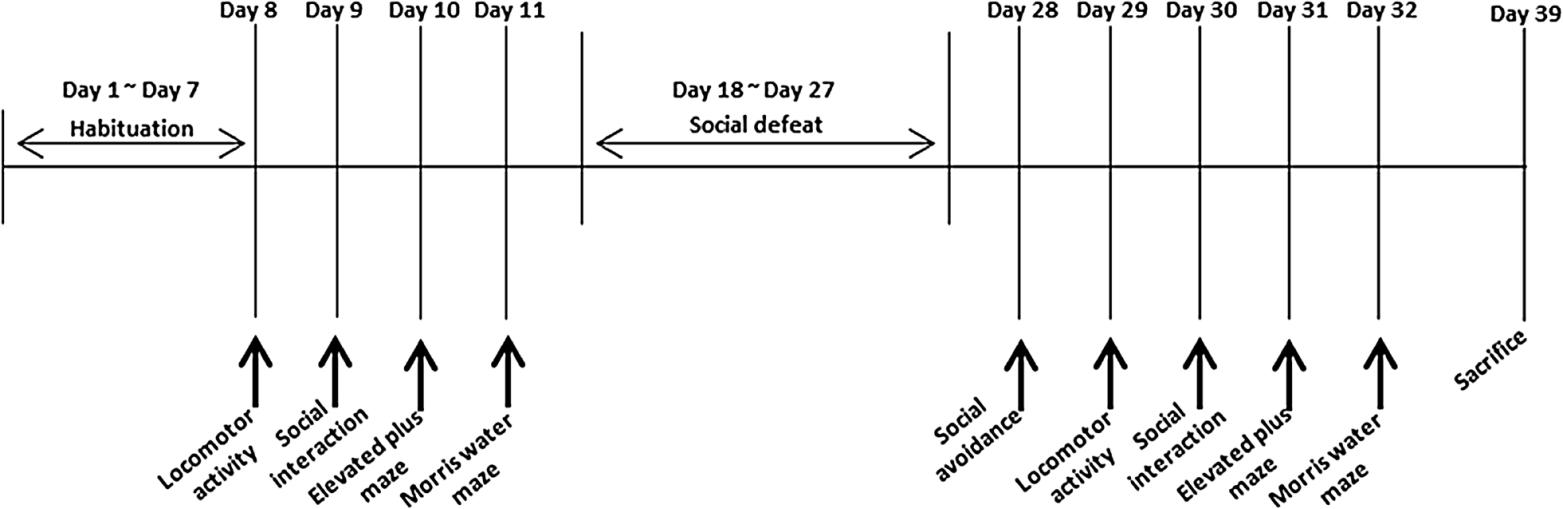
Timeline of the experimental procedures

Following the 1-week habituation period, the behavioral tests were initiated in order of stress intensity (Fig. 1). Next, the C57BL/6N mice were subjected to the chronic social defeat procedure for 10 consecutive days; the defeated mice were categorized into susceptible and unsusceptible groups based on performance in the social avoidance test. Then, the behavioral tests were performed again. On day 39, the mice were sacrificed and brain tissues were obtained for the molecular studies.

### Behavioral tests

All mice were habituated to the behavioral testing room for 30–60 min prior to all behavioral tests. After each behavioral test mice were rested for 1 day.

### Spontaneous locomotor activity

Locomotor activity was measured in an open acrylic box (30 × 40 × 50 cm) using a video tracking system with SMART software (Panlab; Barcelona, Spain). The mice were placed into the testing apparatus and their activities, including distance traveled, locomotion time, and time spent in a central zone (defined as 25% or 50% of the total box area), were recorded for 30 min.

### Social interaction test

The social interaction apparatus consisted of a standard polypropylene rectangular box (30 cm height × 40 cm width × 50 cm length) with an open top in which each C57BL/6N mouse was paired with an unfamiliar CD1 mouse with a similar weight. First, the C57BL/6N mice were habituated to the interaction box for 10 min and then returned to the home cage. Next, after the CD1 mouse was habituated for 10 min, the previously habituated C57BL/6N mouse was reintroduced into the box. Behaviors were recorded for 10 min with two video cameras under dimly lit conditions (40 lx) and the following behaviors were analyzed: (a) dominant behaviors, including upright/sideways offensive posture, attacks or bites, mounting/climbing, aggressive or violent grooming, and tail rattling; (b) submissive behaviors, including upright/sideways defensive posture, crouching, upright/sideways submissive posture, full submission posture, passive anogenital sniffing/being sniffed at the body part, avoidance, and curling up in the corner and remaining motionless; (c) neutral behaviors, including rearing/wall rearing, sniffing at the air or cage, and self-grooming; and (d) social behaviors, including approaching/following, nose sniffing, anogenital sniffing, and social grooming/sniffing. Total time spent and total numbers (frequency) of each behavior (counted when the duration was ≥ 1 s) were scored by an investigator blind to the conditions.

### Elevated plus maze test

The test apparatus for the EPM was constructed from white plexiglas material and included two open arms (35 cm long × 5 cm wide) and two closed arms (35 cm long × 5 cm wide enclosed by 15 cm high walls) that extended from a central platform (5 × 5 cm). The maze was elevated 40 cm above the floor, illuminated at an intensity of 40 lx, and the edges of the open arms were raised 0.25 cm to minimize the chance of a mouse falling. Each mouse was placed in the center facing an open arm and allowed to explore the apparatus for 5 min. Times spent in the open and closed arms and the numbers of open and closed arm entries were calculated; arm entries were defined as entry of all four paws into an arm [34]. If freezing occurred for more than 30% of the total test time (> 100 s), the mouse was excluded from data analysis [35].

### Morris water maze test

The MWM consisted of a circular tank that was 100 cm in diameter and filled with opaque water (23 ± 1 °C) containing non-toxic white paint (Tempera, Dick Blick Holdings, Inc.; Galesburg, IL, USA). A circular escape platform (10 cm in diameter) was hidden 1 cm below the surface of the water. SMART software (Panlab) was used to calculate escape latency, distance traveled, and average swim speed. One day of pre-training (six trials to a fixed visible platform from a fixed start) was performed to assess motor and visual ability and then each animal participated in five trials per day for 5 consecutive days using the submerged platform and extra-maze cues. After end of the trial, wet body of the mouse was dried using towel and returned to its home cage and 1 min interval was given between trials.

When an animal failed to locate the platform within the 90-s time limit, an escape latency of 90 s was assigned. If a mouse floated, it was left alone. If a mouse floated the entire trial, it was removed and given a second trial at a later time. If the same mouse floated in the second trial, it was given up to two trials the next day. If a mouse never searched the maze, it was eliminated from the analyses [36]. Mice with repeated episodes of excessive floating (> 10 s/trial in ≥ 25% of trials) were also excluded from the analyses [37]. Floating was empirically determined as swimming at a speed < 4 cm/s. A different starting position was used for each trial performed on the same day with the sequence of starting positions varying from day to day. On the day after the acquisition training was completed (day 6), a probe test was performed in which the platform was removed and the animals were allowed to search for the maze for 90 s. Time spent and distance traveled in the target quadrant (where the platform had been located) were computed.

### Chronic social defeat stress (CSDS)

The exact procedure for inducing social defeat stress has been described in previous articles from our research group [21, 38]. Briefly, C57BL/6N mice were introduced into the home cage of an unfamiliar CD1 aggressor mouse and they were allowed to interact for 10 min. We intervened to stop serious or prolonged confrontation [39]. During this exposure, all subject mice were defeated and showed signs of subordination (i.e., lying on their backs, freezing, or showing upright submissive postures). The social defeat procedure lasted for 10 consecutive days. We checked the wounds every time after social defeat bout. The mice with wound size greater than 1 cm were supposed to be removed based on the recommendation by Golden et al. [40] but we never saw mice with large wounds. The eight mice with small wounds were treated with betadine and excluded from the experiments. Control group experienced similar experimental conditions. During social defeat stress control mice were housed by pairs in equivalent cages with members of the same strain, one on each side of a perforated plexiglass partition and rotated on daily basis [40]. Based on the results of the social avoidance test, the animals were divided into susceptible and unsusceptible subgroups on day 11.

### Social avoidance test

On 28th day mice were divided into the susceptible and unsusceptible group by performing social avoidance test. The defeated mouse was placed in interaction box (42 × 42 cm) with an empty wire mesh cage (10 × 45 cm) located at the one end. Interaction zone of 8 cm wide area surrounding the wire mesh cage was created. Test performed in two sessions. The first session without target i.e. wire mesh cage is empty and movement of the defeated animal tracked for 2.5 min. There was an interval of 1 min between 2 sessions. In the second session, novel CD1 mouse was introduced into wire mesh cage and the same defeated animal from first session was placed into the box and tracked for another 2.5 min. Mice activity near interaction zone was tracked by automated video tracking system based on the spontaneous motor activity recording tracking (SMART) software (Panlab, Barcelona, Spain). Social interaction (SI) ratio of 100 was set as the cut-off value. The interaction ratio is calculated as 100 × (time spent in the interaction zone with an aggressor)/(time spent in the interaction zone without an aggressor). Mice which scores ≥ 100 were considered as unsusceptible group and mice which scores < 100 were considered as susceptible mice [40].

### Preparation of brain tissue

Approximately 1–2 days after the completion of the behavioral experiments, all animals were killed by cervical dislocation. Brain tissues were collected from the PFC, striatum (ST), AMY, and hippocampus (HIP). An adult mouse brain slicer matrix cooled on ice (BSMAS001-01, Zivic Instruments; Pittsburgh, PA-15237, USA) was used to obtain coronal sections of brain tissues at 1.0 mm intervals. The targeted tissues (PFC, ST, and HIP) were removed from these brain sections on the ice cooled plate using single edge blades and preserved at − 80 °C. The brain slices containing the AMY were immediately cryopreserved using liquid nitrogen and then punched at a later time using a 1.0-mm Harris Uni-Core micro-punch (Electron Microscopy Sciences; Hatfield, PA 19440, USA) in the microtome cryostat (Microm HM 525, Microm international GmbH, part of Thermos Fisher Scientific, Otto-han-str. 1A 69190 Wall Dorf/Germany).

### Western blot analyses

Due to a sufficient number of samples, randomly selected tissues (approximately half) were processed for the Western blot analyses. The tissue samples were homogenized with a radio immunoprecipitation assay (RIPA) cell lysis buffer (1×) containing 150 mM sodium chloride, 1% triton X-100, 1% sodium deoxycholate, 0.1% sodium dodecyl sulfate, 50 mM Tris–HCl, 2 mM EDTA, 1% protease (Sigma-Aldrich Korea Ltd.; Yongin, Korea), and phosphatase inhibitor cocktails (Sigma-Aldrich Korea Ltd.) at pH 7.5 using a Teflon pestle (Vintage Thomas; Philadelphia, PA, USA). The tissue homogenates were subjected to sonication for 5 min (amplitude 20%, on/off cycle as 10 s on and 5 s off) and then centrifuged for 15 min at 14,000 rpm at 4 °C. The resulting supernatant fractions were analyzed to estimate protein concentrations with Bio-Rad Protein assays (Bio-Rad Laboratories; Hercules, CA, USA).

The protein samples (20 µg/lane for ARF6, Rab4, Rab11, and p-DARPP-32 at Threonine 34 [p-DARPP-32 Thr34] and p-DARPP-32 at Threonine 75 [p-DARPP-32 Thr75] and 10 µg/lane for GASP-1 and total DARPP-32) were prepared with 2× Laemmli sample buffer and lysis buffer (1:1 dilution) and boiled for 10 min. The protein samples were separated using either 12% or 10% sodium dodecyl sulfate–polyacrylamide gel electrophoresis (SDS-PAGE) for samples with 20 µg/lane and 10 µg/lane, respectively, and then transferred to a hydrophobic polyvinylidene difluoride (PVDF) membrane; prior to the transfer, the PVDF membranes were treated with methanol for 10 min. The membranes were then blocked with 5% skim milk or 5% bovine serum albumin (BSA) for 2 h at room temperature and incubated overnight at 4 °C with primary antibodies, including mouse monoclonal ARF6 (1:1000, Santa Cruz Biotechnology Inc.; Dallas, Texas, USA), mouse monoclonal Anti-Rab4 and Rab11 (1:1000, BD Transduction Laboratories; Erembodegem, Belgium), rabbit polyclonal GASP-1 (1:1000, Synaptic System; Gottingen, Germany), rabbit monoclonal total DARPP-32 (1:10,000, Epitomics, an Abcam Company; Cambridge, MA 02139-1517, USA), rabbit monoclonal p-DARPP-32 Thr34, and rabbit polyclonal p-DARPP-32 Thr75 (1:1000, Cell Signaling Technology; Denvers, MA, 01923, USA). After washing the membranes three times with Tris-buffered saline (TBS) containing 0.2% Tween 20 (TBST), the primary antibodies were detected using secondary goat anti-mouse IgG-HRP antibodies for ARF6, Rab4, and Rab11 (1:5000, Santa Cruz Biotechnology Inc.) and peroxidase-labeled goat anti-rabbit IgG(H+L) antibodies for GASP-1, total DARPP-32, p-DARPP-32 Thr34, and p-DARPP-32 Thr75 (1:5000, Vector Laboratories; Burlingame, CA, USA) for 2 h at room temperature (25 °C). The density of intracellular trafficking protein bands were normalized to β-actin.

The D2R isoforms (D2L and D2S) were analyzed using a procedure described by McDougall et al. [41] with a few modifications. The protein samples were prepared with 10 µg/lane and sample buffer and lysis buffer (1:1 dilution), kept at room temperature for 1 h, and then separated on 15% gel. The stacking gel was run at 60 V for 30 min during the initial phase, then at 60 V for 30 min until a good separation of the protein markers at 50 kDa was visible and, finally, at 140 V for 150 min. After transfer to the PVDF membranes, they were treated with 0.25% glutaraldehyde for 15 min [42] to improve the signal/noise ratio by decreasing the non-specific binding of secondary antibodies. Next, the glutaraldehyde-treated membrane were washed three times with TBST and blocked with 5% skim milk. The membrane was incubated in 5% skim milk overnight at 4 °C with the synthesized rabbit polyclonal antibodies for D2L (1:2000) and D2S (1:5000) (Abclon Inc. Seoul, Korea). After the membranes were washed three times, the primary antibodies were detected using peroxidase-labeled goat anti-rabbit IgG (H+L) (1:3000 for D2L and 1:5000 for D2S, Vector Laboratories) for 2 h at room temperature (25 °C). Western blot bands were developed using enhanced chemiluminescence reagents (GE Healthcare Inc.; Piscataway, NJ, USA), visualized using the Fusion Solo S imaging system (Vilber Lourmat; Marne-la-Vallee, France), and quantified with a densitometric measurement using Image J software a java based freeware by Wayne Rasband from National Institute of Health, USA. The density of the D2R isoform’s protein bands were normalized to glyceraldehyde-3-phosphate dehydrogenase (GAPDH).

### Synthesis and specificity of antibodies for *D2L and D2S*

Primary antibodies for D2L and D2S were ordered from Abclon Inc. (Seoul, Korea) to obtain subtype-specific staining against both the D2L and D2S isoforms using a procedure described by Khan et al. [43]. Briefly, the D2S peptide TPLKDAAR and the D2L peptide SNGSFPVNRRRM, which correspond to residues 238–245 and 259–270, respectively, were derived from the third cytoplasmic loop of the receptor. The D2S peptide was arranged by adopting four amino acids from each side of the insertion site to differentiate it from D2L. The peptides were coupled to the keyhole limpet hemocyanin (KLH) protein, the peptide/KLH conjugate (100 µg) was emulsified in complete Freund’s adjuvant, and the solution was injected into rabbits for antibody development.

Specificity was tested using blocking peptides: SNGSFPVNRRRM-C (purity 94.86%) and TPLKDAAR-C (purity 92.78%) for D2L and D2S respectively (Abclon Inc. Seoul, Korea). The membranes incubated with blocked antibodies showed no band around 50 kDa markers whereas the membranes treated with control antibodies generated good signals near 50 kDa without non-specific bands surrounding the target protein bands. For the results of antibody specificity test, refer to the Additional file 1.

### Statistical analysis

Outliers were defined as values outside a range of two standard deviations from the mean i.e., Mean ± 2SD of the respective group and were excluded from the analyses. The proportions of outliers were approximately 5–10% for the locomotion and social interaction tests and all Western blot analyses and 20% for the EPM test. For EPM outlier numbers were more than other behavioral tests because we applied one more criteria in which we excluded mice which shown freezing behavior for extended period of time on open arms (time spent on open arms is more than 30% of the total test time i.e., more than 100 s) due to noise or movement by experimenter during testing [35]. The behavioral and Western blot results are presented as a mean ± standard error of the mean (Mean ± S.E.M). For all the data except the frequency of social interacting behaviors, we performed one-way ANOVA. The social interacting behaviors were analyzed by Kruskal–Wallis H test because of skewed distribution. All data were analyzed using SPSS version 21.0 (SPSS Inc.; Chicago, USA). Pearson’s correlation was performed to assess the correlation between SI ratio, and protein expression levels and behavioral data obtained after defeat stress. Correlation plots were constructed using PRISM version 6.0 (GraphPad software, California, USA). In all cases, *p* values < 0.05 were considered to indicate statistical significance.

## Results

### Social defeat stress

During the social defeat procedure (n = 69), eight mice were found to have small wounds. They were all removed from the experiments. The remaining defeated mice (n = 61) exhibited signs of subordination during the attack including fleeing, vocalizing, freezing, showing upright and sideway submissive postures, and lying on the back and exposing the belly to the attacker. Following this procedure, 65.6% of mice were classified as susceptible (n = 40) and 34.4% were classified as unsusceptible (n = 21).

### Spontaneous locomotor activity

Following the social defeat procedure, the distances traveled and locomotion times significantly decreased compared to baseline (i.e., prior to social defeat) in both the unsusceptible (*p *= 0.001 and *p *< 0.001, respectively) and susceptible (*p *= 0.018 and *p *= 0.026, respectively) groups (Table 1). Times spent in the central zone also significantly decreased in all three groups but a group comparison of the change values revealed that only locomotion time significantly differed among the groups (F_[2, 70]_ = 8.023, *p *= 0.001). Post hoc analyses revealed significant differences between the unsusceptible and control groups (*p *= 0.001) and susceptible and unsusceptible groups (*p *=0.004).

**Table 1:** Comparison of locomotor activities obtained before and after social defeat stress among three groups

Parameters	Control group (n = 15)			*p* ^a^	Unsusceptible group (n = 20–21)			*p* ^a^	Susceptible group (n = 38–39)			*p* ^a^	*p* ^b^
	Before	After	Change		Before	After	Change		Before	After	Change		
Distance travelled (cm)	10,265.16 ± 813.84	9520.58 ± 635.32	− 593.49 ± 812.32	0.401	9416.40 ± 565.47	7138.97 ± 483.44	− 1457.57 ± 617.62	0.001	9082.38 ± 296.26	8313.24 ± 292.81	− 629.96 ± 317.99	0.018	0.431
Time spent in central zone (25%)	83.87 ± 17.63	27.24 ± 4.96	− 49.88 ± 9.92	0.004	104.49 ± 15.02	51.70 ± 7.66	− 57.66 ± 13.48	0.002	103.65 ± 11.94	28.54 ± 5.23	− 67.35 ± 7.88	< 0.001	0.494
Time spent in central zone (50%)	357.77 ± 65.91	158.46 ± 31.14	− 154.02 ± 33.75	0.008	335.34 ± 36.32	134.81 ± 24.06	− 177.15 ± 33.68	< 0.001	296.35 ± 30.94	152.84 ± 20.38	− 125.55 ± 25.92	< 0.001	0.449
Locomotion time (sec)	1205.35 ± 78.34	1121.15 ± 62.03	− 41.86 ± 65.94	0.347	1138.77 ± 56.74	841.68 ± 65.19	− 330.28 ± 59.17*^†^	< 0.001	1123.74 ± 33.28	1038.77 ± 29.33	− 116.99 ± 32.72	0.026	0.001

Data expressed as mean ± S.E.M

^a^Student’s paired t test between the data obtained before and after social defeat stress

^b^One-way ANOVA for the change

* *p *< 0.05 versus susceptible group

^†^*p *< 0.05 versus control group

### EPM test

**Fig. 2:**
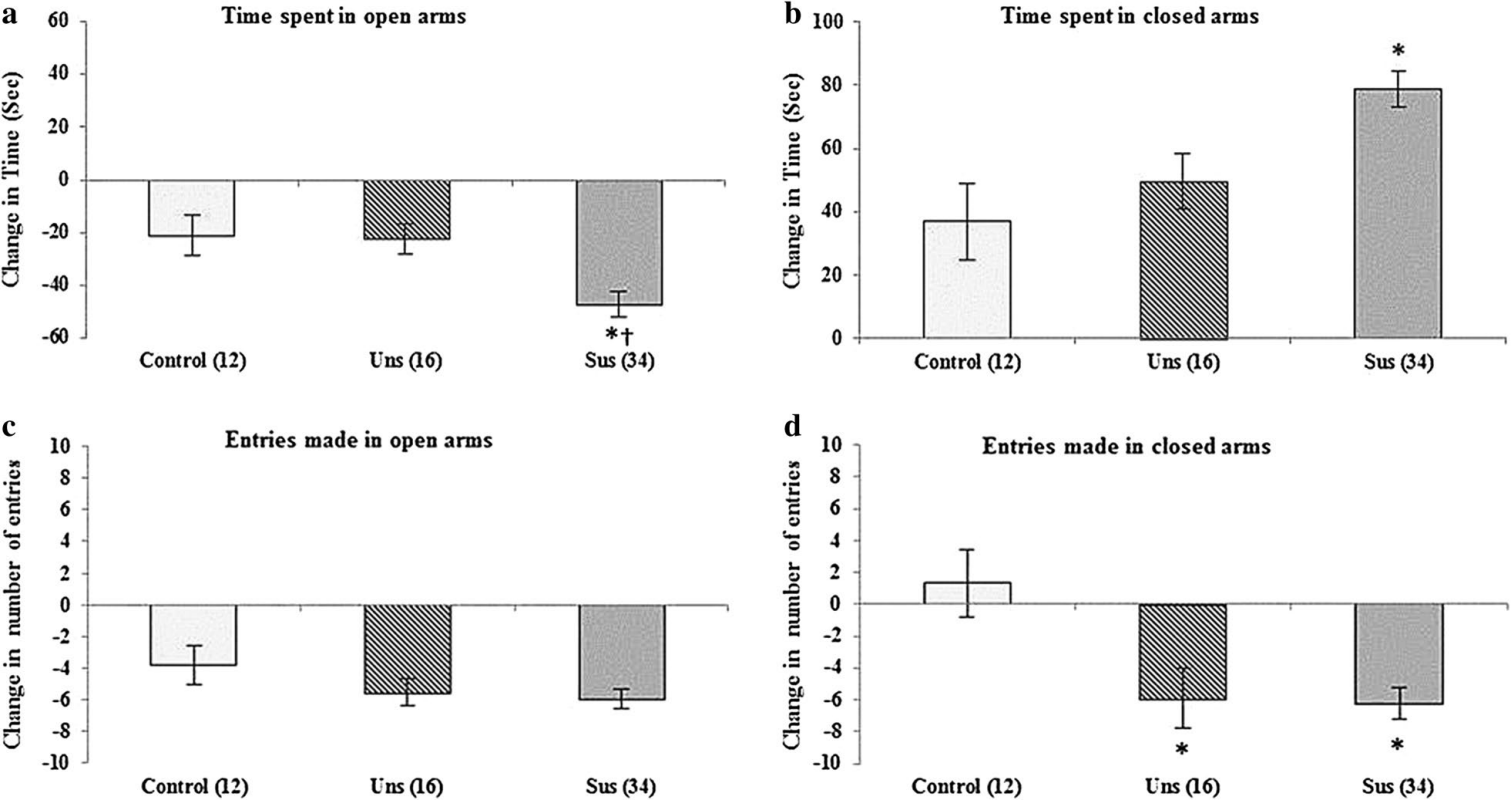
Anxiety-like behavioral change profiles were tested by elevated plus maze test and compared among three groups. *p < 0.05 versus control group; ^†^*p *< 0.05 versus unsusceptible group

Following the social defeat procedure, time spent in the open arms and number of entries into the open arms significantly decreased compared to baseline in all three groups (Table 2 and Fig. 2). Time spent in the closed arms increased in all three groups whereas the number of entries into the closed arms decreased in the unsusceptible and susceptible groups. A group comparison of the change values revealed that times spent in the open (F_[2, 59]_ = 6.884, *p *= 0.002) and closed (F_[2, 59]_ = 7.252, *p *= 0.002) arms and the number of entries into the closed arms (F_[2, 59]_ = 6.866, *p *= 0.002) significantly differed among the groups. The post hoc analyses revealed that the change in time spent in the open arms in the susceptible group was greater than those in the control (*p *= 0.009) and unsusceptible (*p *= 0.017) groups and that the change in time spent in the closed arms in the susceptible group was greater than that in the control group (*p *= 0.003). Additionally, the number of entries into the closed arms in the susceptible (*p *= 0.003) and unsusceptible (*p *= 0.013) groups were greater than those of the control group.

**Table 2:** Comparison of the results with elevated plus maze test obtained before and after social defeat stress among three groups

Parameters	Control group (n = 12)			*p* ^a^	Unsusceptible group (n = 16)			*p* ^a^	Susceptible group (n = 34)			*p* ^a^	*p* ^b^
	Before	After	Change		Before	After	Change		Before	After	Change		
Time spent in arms (s)													
Open arm	39.90 ± 6.36	18.89 ± 4.90	− 21.01 ± 7.72	0.020	32.77 ± 4.70	10.38 ± 3.08	− 22.38 ± 5.83	0.003	53.12 ± 4.95	5.93 ± 1.66	− 47.18 ± 4.97*^†^	< 0.001	0.002
Closed arm	212.43 ± 8.54	249.29 ± 9.48	36.85 ± 12.04	0.011	221.55 ± 6.68	270.98 ± 4.72	49.42 ± 8.79	< 0.001	199.25 ± 5.28	277.99 ± 2.83	78.74 ± 5.84*	< 0.001	0.002
Number of entries													
Open arm	6.16 ± 1.02	2.33 ± 0.52	− 3.83 ± 1.19	0.008	7.09 ± 1.54	1.54 ± 0.65	− 5.54 ± 0.85	< 0.001	7.00 ± 0.55	1.03 ± 0.24	− 5.96 ± 0.58	< 0.001	0.275
Closed arm	14.91 ± 1.00	16.25 ± 1.23	1.33 ± 2.11	0.541	16.90 ± 1.58	11.0 ± 1.09	− 5.90 ± 1.86*	0.010	15.5 ± 0.61	9.25 ± 0.78	− 6.25 ± 0.99*	< 0.001	0.002

Data expressed as mean ± S.E.M

^a^Student’s paired t test between the data obtained before and after social defeat stress

^b^One-way ANOVA for the change

* *p *< 0.05 versus control group

^†^*p *< 0.05 versus unsusceptible group

### Social interaction test

**Fig. 3:**
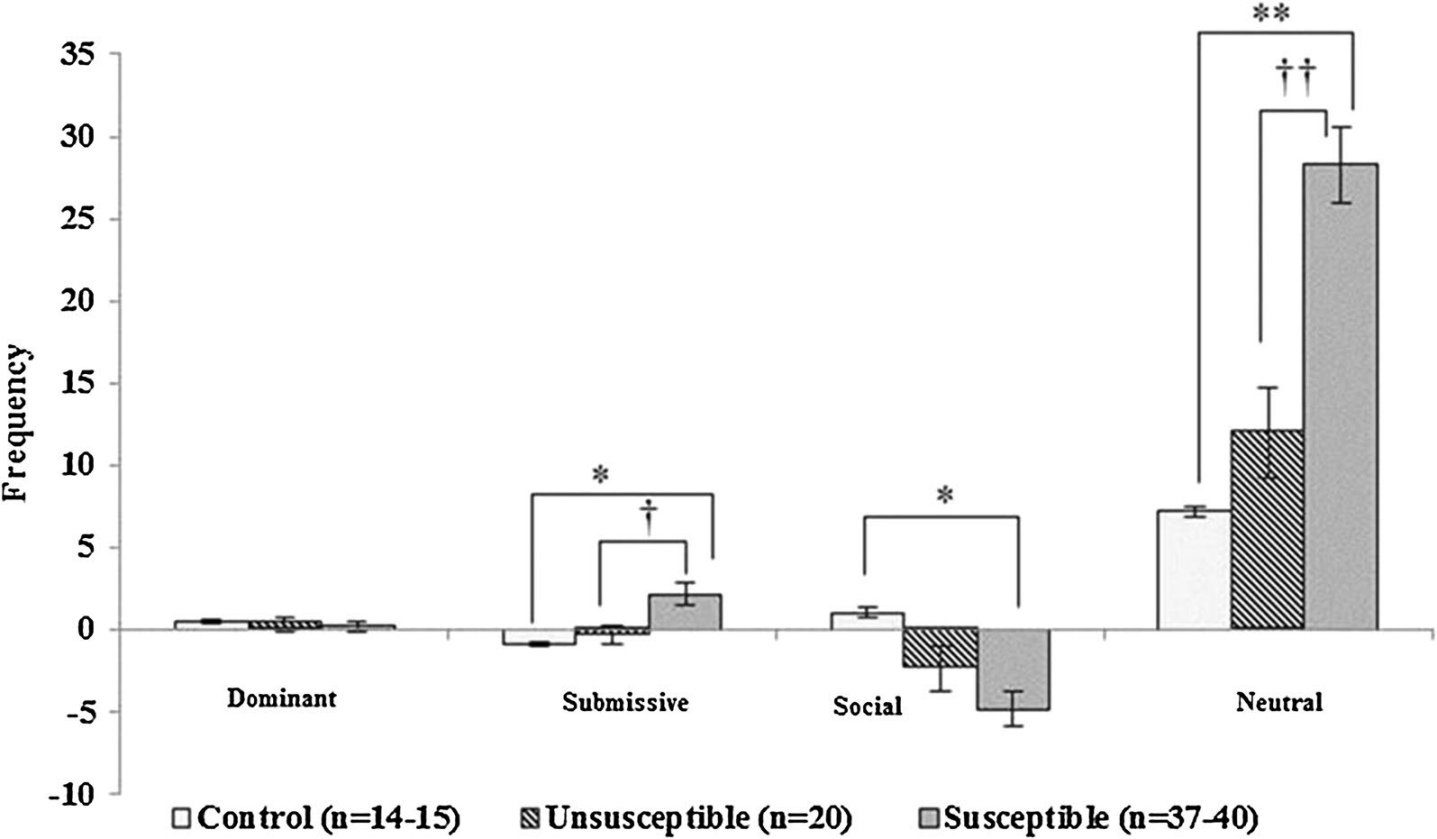
Comparison of the changes in behavior frequencies obtained before and after social defeat stress in social interaction test among three groups. Data are total number (frequency) of each behavioral type and expressed as mean ± S.E.M, *p < 0.05, **p < 0.001 versus control group; ^†^*p *< 0.05, ^††^*p *< 0.001 versus unsusceptible group by Kruskal–Wallis test

A group comparison of the change values revealed significant differences in submissive (F_[2, 68]_ = 5.771, *p *= 0.005), social (F_[2, 70]_ = 5.509, *p *= 0.006), and neutral (F_[2, 72]_ = 19.830, *p *= < 0.001) behaviors among the groups (Fig. 3). The post hoc analyses revealed that the changes in submissive and neutral behaviors in the susceptible group were greater than those in the control (*p *= 0.017) and unsusceptible (*p *= 0.026) groups and that the change in social behaviors in the susceptible group was greater than in the control group (*p *= 0.005). In terms of time spent performing the behaviors, similar patterns of social and neutral behaviors were observed for all groups.

### MWM test

Within- and between-group comparisons of the change values revealed that there were no significant differences in escape latency or path length.

### Dopaminergic marker proteins

#### D2 isoforms

**Fig. 4:**
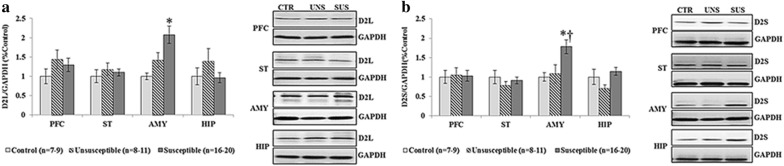
Western blot results of dopamine D2 receptor isoforms among three groups. **a** Comparison of D2L expression levels in the prefrontal cortex (PFC), striatum (ST), amygdala (AMY), and hippocampus (HIP) among three groups, *p < 0.05 versus control group; **b** comparison of D2S expression levels in the PFC, ST, AMY, and HIP among three groups *p < 0.05 versus control group; ^†^*p *< 0.05 versus unsusceptible group. *CTR* control, *UNS* unsusceptible, *SUS* susceptible

There were significant differences in the expression of D2L (F_[2, 32]_ = 5.970, *p *= 0.006) and D2S (F_[2, 33]_ = 5.035, *p *= 0.009] in the AMY among the three groups (Table 3, Fig. 4). The post hoc analyses revealed a significant increase in the expression of D2L in the susceptible group (*p *= 0.008) compared to the control group. The susceptible group also exhibited a significant increase in the expression of D2S compared to the control (*p *= 0.030) and unsusceptible (*p *= 0.044) groups.

**Table 3:** Western blot results of dopamine D2 receptor isoforms and total- and p-DARPP-32 among three groups

	Brain regions	Control group (n = 7–9)	Unsusceptible group (n = 8–11)	Susceptible group (n = 16–20)	*p*
D2L	PFC	1 ± 0.19	1.44 ± 0.24	1.29 ± 0.17	0.453
	ST	1 ± 0.16	1.18 ± 0.15	1.10 ± 0.09	0.695
	AMY	1 ± 0.17	1.17 ± 0.16	1.65 ± 0.16*	0.006
	HIP	1 ± 0.22	1.39 ± 0.32	0.96 ± 0.13	0.307
D2S	PFC	1 ± 0.16	1.04 ± 0.19	1.02 ± 0.14	0.988
	ST	1 ± 0.17	0.77 ± 0.10	0.91 ± 0.08	0.503
	AMY	1 ± 0.11	1.08 ± 0.23	1.77 ± 0.17*^†^	0.009
	HIP	1 ± 0.19	0.70 ± 0.09	1.14 ± 0.09	0.058
Total DARPP-32	PFC	1 ± 0.14	1.38 ± 0.16	1.16 ± 0.11	0.246
	ST	1 ± 0.15	0.81 ± 0.13	0.88 ± 0.11	0.719
	AMY	1 ± 0.18	1.23 ± 0.14	0.93 ± 0.08	0.191
	HIP	1 ± 0.28	1.02 ± 0.17	0.60 ± 0.06	0.071
p-DARPP-32 Thr34	PFC	1 ± 0.07	1.15 ± 0.12	1.33 ± 0.09	0.113
	ST	1 ± 0.11	1.15 ± 0.14	0.98 ± 0.07	0.475
	AMY	1 ± 0.14	1.31 ± 0.14	1.42 ± 0.13	0.185
	HIP	1 ± 0.23	0.93 ± 0.13	0.86 ± 0.07	0.781
p-DARPP-32 Thr75	PFC	1 ± 0.08	1.20 ± 0.11	1.09 ± 0.06	0.349
	ST	1 ± 0.14	0.97 ± 0.09	0.96 ± 0.07	0.976
	AMY	1 ± 0.02	0.70 ± 0.02*	0.77 ± 0.04*	0.002
	HIP	1 ± 0.13	1.11 ± 0.13	1.05 ± 0.13	0.899

Data were expressed in mean ± S.E.M

* *p *< 0.05 versus control group

^†^*p *< 0.05 versus unsusceptible group

#### Darpp-32

Of the DARPP-32 proteins, only the level of p-DARPP-32 Thr75 in the AMY significantly differed among the three groups [F_(2, 35)_ = 7.406, *p *= 0.002]. Post hoc analyses revealed a significant increase in the expression of p-DARPP-32 Thr75 in the unsusceptible (*p *= 0.002) and susceptible (*p *= 0.008) groups compared to the control group.

### Intracellular trafficking-related proteins

**Fig. 5:**
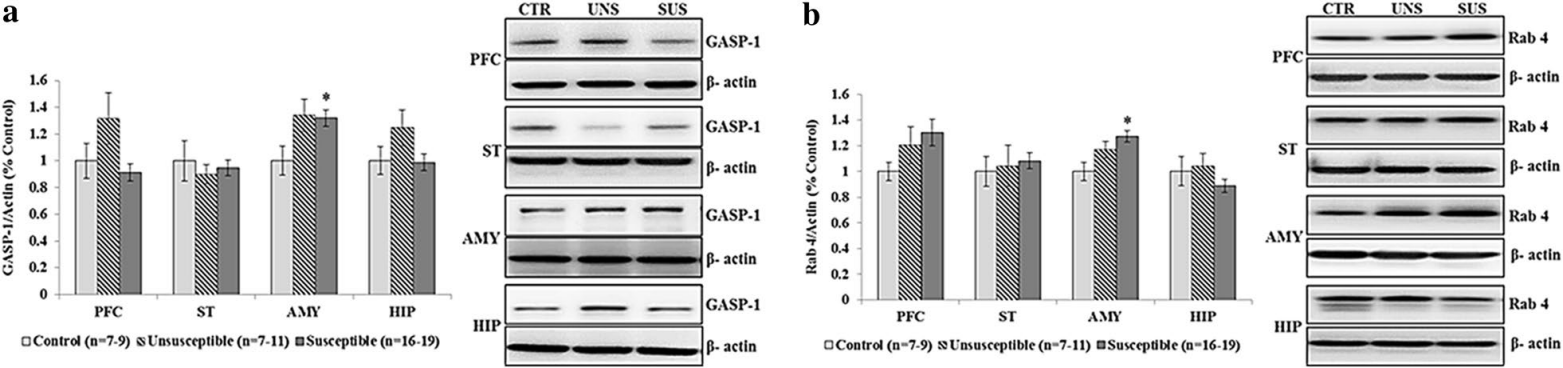
Western blot results of GASP-1 and Rab4 among three groups. **a** Comparison of GASP-1 expression levels in the prefrontal cortex (PFC), striatum (ST), amygdala (AMY), and hippocampus (HIP) among three groups, *p < 0.05 versus control group; **b** comparison of Rab4 expression levels in the PFC, ST, AMY, and HIP among three groups *p < 0.05 versus control group. *CTR* control, *UNS* unsusceptible, *SUS* susceptible

A one-way ANOVA revealed significant differences in the levels of Rab4 (F_[2, 34]_ = 6.126, *p *= 0.005) and GASP-1 (F_[2, 37]_ = 3.435, *p *= 0.043) in the AMY among the three groups (Table 4 and Fig. 5). Post hoc analyses revealed that the expression of Rab4 (*p *= 0.004) and GASP-1 (*p *= 0.048) exhibited significant increases in the susceptible group compared to the control group.

**Table 4:** Western blot results of intracellular trafficking related proteins (ARF-6, GASP-1, Rab4 and Rab11) among three groups

	Brain regions	Control group (n = 7–9)	Unsusceptible group (n = 7–10)	Susceptible group (n = 16–19)	*p*
ARF-6	PFC	1 ± 0.13	0.87 ± 0.12	0.99 ± 0.04	0.552
	ST	1 ± 0.12	1.15 ± 0.03	0.95 ± 0.08	0.371
	AMY	1 ± 0.09	1.37 ± 0.09	1.20 ± 0.08	0.094
	HIP	1 ± 0.12	0.95 ± 0.11	1.06 ± 0.07	0.691
GASP-1	PFC	1 ± 0.13	1.31 ± 0.19	0.91 ± 0.06	0.054
	ST	1 ± 0.14	0.89 ± 0.07	0.94 ± 0.05	0.249
	AMY	1 ± 0.10	1.33 ± 0.12	1.32 ± 0.06*	0.043
	HIP	1 ± 0.10	1.24 ± 0.13	0.98 ± 0.06	0.123
Rab4	PFC	1 ± 0.07	1.20 ± 0.14	1.30 ± 1.10	0.276
	ST	1 ± 0.11	1.04 ± 0.16	1.08 ± 0.06	0.944
	AMY	1 ± 0.07	1.17 ± 0.06	1.27 ± 0.04*	0.005
	HIP	1 ± 0.11	1.04 ± 0.09	0.88 ± 0.04	0.303
Rab11	PFC	1 ± 0.06	1.17 ± 0.08	1.21 ± 0.04	0.097
	ST	1 ± 0.12	1.22 ± 0.05	1.17 ± 0.06	0.204
	AMY	1 ± 0.10	1.07 ± 0.09	1.07 ± 0.04	0.742
	HIP	1 ± 0.13	1.20 ± 0.09	1.21 ± 0.04	0.185

Data were expressed in mean ± S.E.M

*ARF-6* ADP-ribosylation factor 6, *GASP-1* GPCR associated sorting protein-1

* *p *< 0.05 versus control group

### Correlation analysis

**Fig. 6:**
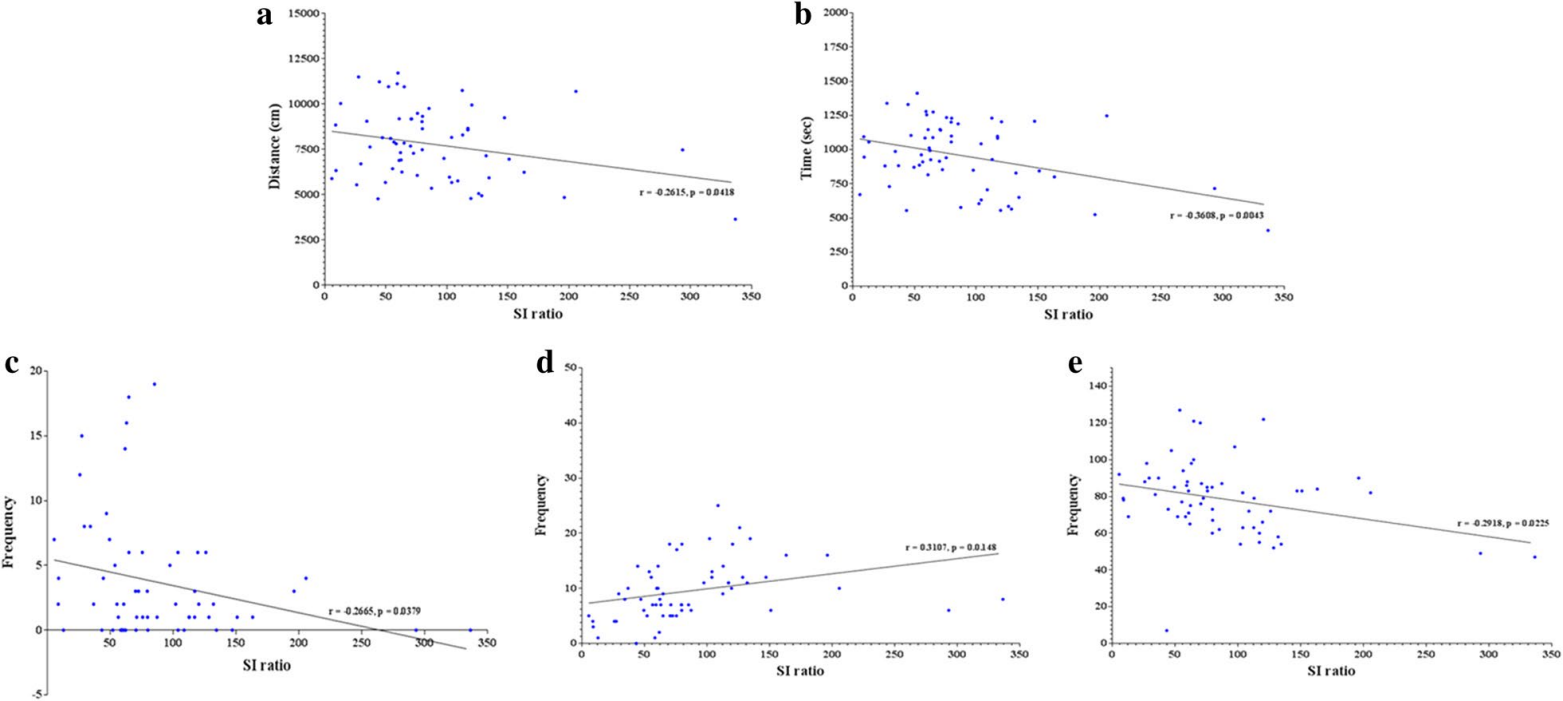
Correlations between social interaction (SI) ratio and behaviors (**a** distance travelled; **b** locomotion time; **c** submissive behaviors; **d** social behaviors; and **e** neutral behaviors) in defeated mice (n = 61)

Significant negative correlations were observed between SI score, and distance travelled (*r* = − 0.2615; *p *= 0.0418), locomotion time (*r* = − 0.3608; *p *= 0.0043), and submissive (*r* = − 0.2665; *p *= 0.0.0379) and neutral (*r* = − 0.2918; *p *= 0.0225) behaviors (Fig. 6a–c, e). Significant positive correlation was found with social (*r* = 0.3107; *p *= 0.0148) behavior (Fig. 6d).

**Fig. 7:**
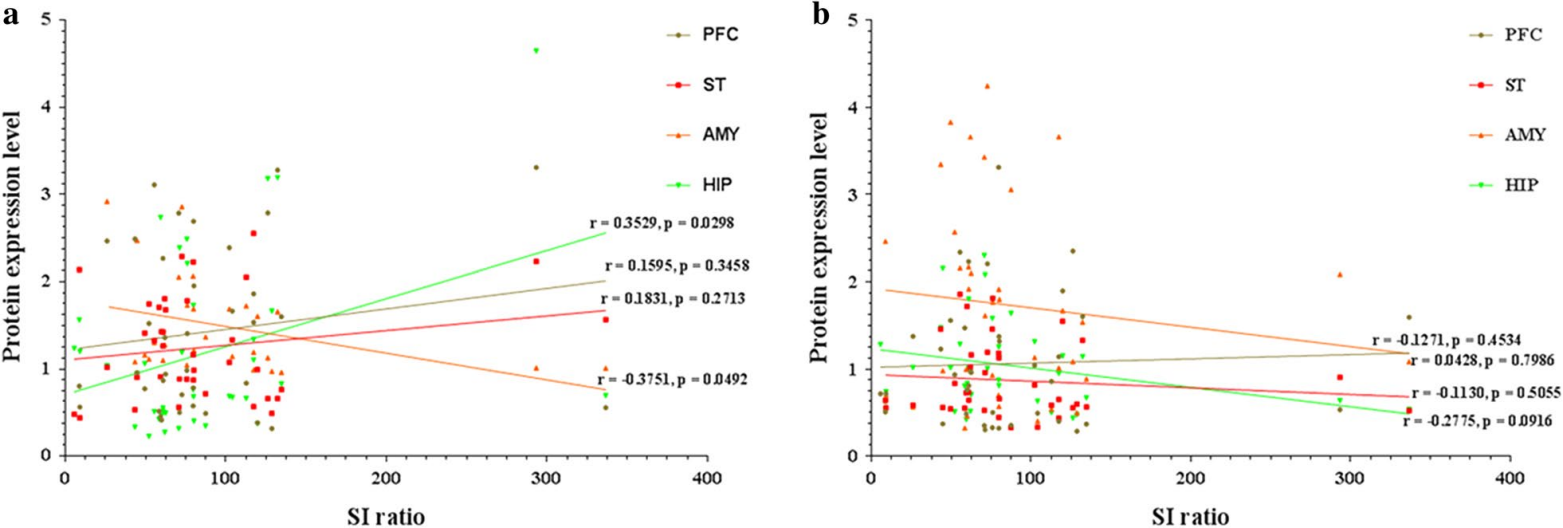
Correlation plots depicting relationship between social interaction (SI) ratio and expression level of dopamine receptor isoforms in the prefrontal cortex (PFC), striatum (ST), amygdala (AMY) and hippocampus (HIP) (n = 31): **a** SI ratio vs. D2L; and **b** SI ratio vs D2S

**Fig. 8:**
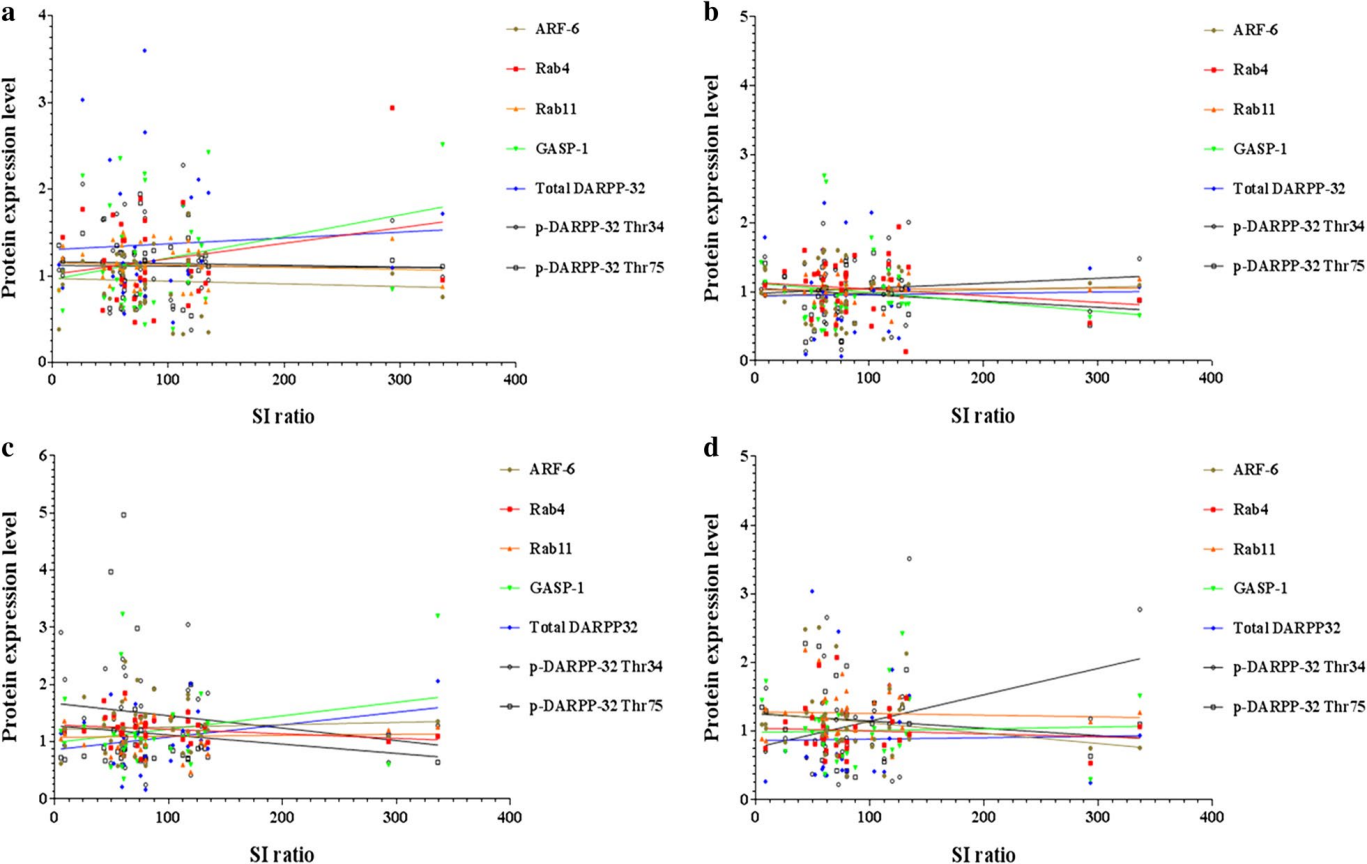
Correlation plots depicting relationship between social interaction (SI) ratio and expression level of intracellular trafficking proteins: **a** for the PFC, SI ratio vs. ARF-6 (r = − 0.0621, *p* = 0.7108), Rab4 (r = 0.2345, *p* = 0.2041), Rab11 (r = − 0.0684, *p* = 0.6831), GASP-1 (r = 0.2689, *p* = 0.1026), total DARPP-32 (r = 0.0628, *p* = 0.7241), p-DARPP-32 Thr34 (r = − 0.0101, *p* = 0.9516), and p-DARPP-32 Thr75 (r = − 0.0387, *p* = 0.8199); **b** for the ST, SI ratio vs. ARF-6 (r = 0.06631, *p* = 0.6966), Rab4 (r = − 0.1559, *p* = 0.3712), Rab11 (r = 0.01701, *p* = 0.9193), GASP-1 (r = − 0.1771, *p* = 0.2873), Total DARPP-32 (r = 0.02249, *p* = 0.9061), p-DARPP-32 Thr34 (r = 0.1018, *p* = 0.5432), and p-DARPP-32 Thr75 (r = − 0.1627, *p* = 0.3359); **c** for the AMY, SI ratio vs. ARF-6 (r = 0.6010, *p* = 0.7159), Rab4 (r = − 0.2099, *p* = 0.2570), Rab11 (r = 0.0396, *p* = 0.8131), GASP-1 (r 0.2430 = , *p* = 0.1416), total DARPP-32 (r = 0.3319, *p* = 0.0552), p-DARPP-32 Thr34 (r = − 0.2107, *p* = 0.2316), and p-DARPP-32 Thr75 (r = -0.1134, *p* = 0.4980); **d** for the HIP, SI ratio vs. ARF-6 (r = − 0.1926, *p* = 0.2605), Rab4 (r = − 0.0774, *p* = 0.6789), Rab11 (r = − 0.0474, *p* = 0.7802), GASP-1 (r 0.0400 = , *p* = 0.8115), Total DARPP-32 (r = 0.0208, *p* = 0.9114), p-DARPP-32 Thr34 (r = 0.3428, *p* = 0.0472), and p-DARPP-32 Thr75 (r = − 0.1454, *p* = 0.4786)

As for D2 isoforms, we observed negative and positive correlation between SI ratio and D2L expression levels in the AMY (*r* = − 0.3751; *p *= 0.0492) and HIP (*r* = 0.3529; *p *= 0.0298*),* respectively (Fig. 7a). On the other hand, no significant correlations were found between SI ratio and D2S in any of the brain regions (Fig. 7b). As for intracellular trafficking proteins, only p-DARPP-32 Thr34 (*r* = 0.3428; *p *=0.0472) in the HIP was positively correlated with SI ratio (Fig. 8d).

## Discussion

The present study investigated the effects of social defeat stress on a variety of behavioral parameters, including the social interaction, EPM, and MWM tests, and assessed the levels of various dopaminergic markers (D2L, D2S, and total and p-DARPP-32) and proteins involved in intracellular trafficking in several key brain regions of C57BL/6N mice. Following the social defeat procedure, there were significant changes in behavior during the EPM and social interaction tests and significant alterations in the expression levels of D2L, D2S, p-DARPP-32 Thr75, Rab4, and GASP-1 in the AMY of the susceptible and/or unsusceptible groups compared to the control group.

### Locomotor activity and anxiety-like behaviors in response to CSDS

In the spontaneous locomotor activity test, only the change in locomotion time after social defeat was greater in the unsusceptible group compared to the control and susceptible groups. These findings are consistent with those of previous reports [11, 44] but these studies did not classify the subjects into susceptible and unsusceptible subpopulations. On the other hand, Krishnan et al. [9] reported no change in locomotor activity in both susceptible and unsusceptible mice compared to controls. This discrepancy may be due to methodological differences between the studies. More specifically, the present study compared changes from baseline among three groups while Krishnan et al. [9] compared behavior among three groups only after social defeat. The present study found that locomotion time significantly decreased after social defeat in both the susceptible and unsusceptible groups but the degree of change was greater in the unsusceptible group. The observed negative correlation results with SI ratio suggest that susceptible mice are more hyperactive compared to unsusceptible mice. This study also showed that there were greater decrease in time spent in the open arm and greater increase in time spent in the closed arm in the susceptible group compared to the control group and greater decreases in the number of entries into the closed arms in the susceptible and unsusceptible groups compared to the control group in the EPM test. These findings are consistent with those of Krishnan et al. [9] and a study showing that C57BL/6J male mice defeated by a conspecific display fewer open and total entries than controls [45]. Various environmental stressors, including prolonged isolation, foot shock, and forced swim, increase behavioral indices of anxiety in the EPM test [46, 47]. Therefore, the present EPM data indicate that social defeat stress increased anxiety-like behaviors in all defeated mice and that the degree of change was greater in the susceptible group. It was interesting to see decreases of the several parameters in control mice suggesting increased anxiety. This is in same line with Espezo study [48] that anxiety is enhanced after test repetition. Alternatively, it may be due to reduced novelty in the second test.

### Social interaction in response to CSDS

In the social interaction test, there were greater increases in submissive and neutral behaviors and greater decreases in social behaviors from baseline in the susceptible group compared to the control group, which is similar with the findings of previous studies from our research group [38, 49]. However, the present results are unique in that the behavioral parameters were measured twice, before and after social defeat stress, in each group and interaction was performed with an unfamiliar CD1 mouse, not the same genetic background. The greater increase in submissive behaviors in the susceptible group compared to the control and unsusceptible groups was interesting as it could be regarded as an indicator of susceptibility. However, it is also possible that the development of subordinate behaviors is a more adaptive and flexible behavioral strategy [50]. This issue needs to be addressed in future studies. The correlation results reflect the same profile, i.e., lesser social behaviors and greater neutral and submissive behaviors in susceptible mice. We performed the MWM test to evaluate whether CSDS affects spatial learning and memory in defeated mice. No significant findings in the MWM test are similar to the results of previous study [10].

### Dopaminergic marker protein: D2 receptor isoforms

The present analyses of dopaminergic marker proteins showed that the expression of D2L and D2S increased in the AMY of the susceptible group compared to the control and unsusceptible groups. These findings are consistent with those of our previous report [21] except that the previous study also found significant changes in the PFC. The discrepancy may be due to delayed timing of sacrifice in the present study compared to the timing of the previous study in which animals were killed immediately after social avoidance test. The mechanisms underlying the increased expression of D2L and D2S in the AMY in susceptible mice remain unknown but it has been shown that conditioned fear stress enhances dopamine release in the AMY [51]. Because D2Rs are expressed at significant levels in the AMY [52] and the role of presynaptic D2S receptors is to inhibit dopamine release [53], the increased expression of D2S in the AMY of susceptible mice may be a compensatory mechanism that reduces dopamine release induced by social defeat stress. Assuming that released dopamine is more likely to bind to D1Rs, which are several times more abundant in the AMY than D2Rs [53], it could lead to the over activation of adenylyl cyclase. Subsequently, the increased expression of D2L may occur to cope with the over activation of adenylyl cyclase because D2R activation inhibits adenylyl cyclase. The AMY is usually maintained under the control of the medial prefrontal cortex but under the pressure of environmental threats, dopaminergic neurotransmission restores its activity allowing the development of anxiety responses [54, 55]. Accumulating evidence indicates that the mesoamygdaloid dopamine pathway modulates fear and anxiety by innervating preferentially GABAergic interfaces controlling the main input and output of the AMY [56]. More specifically, it has also been suggested that dopamine D1 receptor (D1R) may participate in danger recognition facilitating conditioned–unconditioned associations by retrieving the affective properties of the unconditioned environmental stimuli while D2R may instead participate in the modulation of reflex-like behaviors organized in the brain stem and in the setting up of adaptive responses to cope with aversive environmental situations [56]. Hence, our findings on D2S and D2L expressions in the AMY, and social interaction test indicate that susceptible mice are more likely to perceive defeat stress as threatening and in greater need to cope with aversive situations. The correlation analysis shows similar finding for D2L but different for D2S which should be compared cautiously to the results by one-way ANOVA because of no SI ratio for control group in the correlation.

### Dopaminergic marker protein: p-DARPP-32 Thr75

In the present study, only p-DARPP-32 Thr75 expression was found to be significantly decreased in the AMY of defeated mice (susceptible and unsusceptible groups) compared to the control group. Enhanced dopamine states induced by amphetamine or cocaine increase the activity of protein kinase A (PKA) and lead to increases in p-DARPP-32 Thr34 but decreases in p-DARPP-32 Thr75 [57]. Assuming that social defeat stress may increase the release of dopamine in the AMY, the present results are partially consistent with earlier studies that used cocaine or amphetamine [57] suggesting defeated mice are under high dopamine state. Despite the important potential role of DARPP-32 in neuropsychiatric disorders, few studies have investigated the roles of these proteins. In animal studies, increases in total DARPP-32 expression are induced by calorie restriction [58], electroconvulsive stimulation [25], and the inhibitory avoidance task [24]. On the other hand, the expression of DARPP-32 exhibits decreases in the post-mortem brain of patients with schizophrenia [59] and in the leukocytes of patients with schizophrenia and bipolar disorder [60]. Ours is the first report on the levels of p-DARPP-32 in relation to social defeat stress. The correlation analysis shows different findings which should be compared cautiously to the results by one-way ANOVA because of no SI ratio for control group in the correlation.

### Intracellular trafficking protein measures (Rab4 and GASP-1)

The present study also assessed trafficking-related proteins and observed increased expression of Rab4 and GASP-1 in the AMY of the susceptible group compared to the control group. Li et al. [29] demonstrated that there are two D2R recycling pathways that play distinct roles in determining D2R function: the Rab4-sensitive constitutive D2R recycling pathway determines the steady-state surface expression levels of D2Rs whereas the Rab11-sensitive dopamine activity-dependent D2R recycling pathway is important for the functional resensitization of D2Rs. Moreover, acute stress increases the expression of Rab4 and subsequent trafficking processes in rats [61]. Therefore, the present findings that social defeat stress increased the expression of Rab4 in the AMY of susceptible mice may be associated with an increase in the recycling of internalized D2Rs induced by a high dopamine state, which, in turn, would lead to increased D2L levels in the cell membrane. However, this is pure speculation and needs to be confirmed in membrane proteins extracted from subcellular fractionation samples (postsynaptic density fraction) rather than total cell proteins, such as in the present study. GASP-1 is a recently discovered sorting protein for GPCRs that seems to be involved in directing internalized GPCRs to lysosomes for degradation [62]. Hence, the increased expression of GASP-1 observed in the present study may reflect an increased demand for the degradation of internalized D2Rs due to the high dopamine state. The physiological relevance of Rab4 and GASP-1 in terms of D2R expression in the cell membrane should be explored further.

Taken together of our findings, it may be inferred that defeated mice may be under high dopamine state especially in the AMY and increased expression of D2R isoforms seems compensatory mechanism. These changes may be associated with increased anxiety-like behaviors and decreased social behaviors of defeated mice. As negative symptoms including decreased social behaviors are known to be associated with hypo dopaminergic state in the limbic areas [63, 64], this speculation seems counterintuitive. However, considering the report that social withdrawal, a core feature of negative symptoms, is differentiated into passive social withdrawal (PSW) and active social avoidance (ASA) which are associated with negative and positive symptoms respectively [65], decreased social behaviors in the present study may reflect ASA rather than PSW. In light of the demonstrated role of inflammation in behavioral and neuronal phenotypes, it would be interesting to measure inflammatory markers and compare levels of inflammation between susceptible, unsusceptible and control mice in future studies.

### Limitations

The present study has several limitations that should be mentioned. First, although two separated bands were consistently observed at about 48 and 52 kDa, using the protocol adopted from McDougall et al. [41] with subtype-specific antibodies synthesized from Abclon, Inc. (#1403, Ace Twin Tower1, 285 Digital-ro Guro-gu, Seoul 152-779, Korea) Western blot analyses of recombinant Sf9 cell lines expressing D2L and D2S should be carried out to test the specificity and selectivity of the antibodies to D2S and D2L. Second, the Western blot results may not reflect true changes induced by social defeat stress because the animals were exposed to another stressful test, MWM test and sacrificed 12 days after the social defeat stress; this issue should be considered when designing future studies. Third, to explore abnormalities in trafficking caused by social defeat stress, it is desirable to use subcellular fractionation samples rather than whole protein samples as in the present study. Despite these shortcomings, the present study assessed the behavioral parameters twice (before and after social defeat stress), identified D2R isoforms with subtype-specific antibodies, and included a relatively large number of mice in the susceptible and unsusceptible groups.

## Conclusion

This study showed that (i) social defeat stress induces anxiety-like behaviors in spontaneous locomotor activity test or EPM test in defeated mice, (ii) altered submissive, social, and neutral behaviors in susceptible mice, and (iii) altered expression levels of D2 receptor isoforms (D2L and D2S) and intracellular trafficking proteins like Rab4 and GASP-1 in AMY brain region of susceptible mice. Taken together, these results suggest that social defeat stress induce changes in social behaviors and dopaminergic marker proteins which are closely related with pathogenesis of schizophrenia.

## Additional file

**Additional file 1: Figure S1.** Additional western blot results showing band of interest (D2L). **Figure S2.** Additional western blot results showing band of interest (D2S).

## Abbreviations

EPM: elevated plus maze
MWM: Morris water maze
D1R: D1 receptor
D2R: D2 receptor
D2L: D2 long form
D2S: D2 short form
ARF-6: ADP ribosylation factor-6
GASP-1: G protein-coupled receptor (GPCR) associated sorting protein-1
DARPP-32: dopamine and cyclic adenosine 3′,5′-monophosphate-regulated phosphoprotein-32
p-DARPP-32 Thr34 and Thr75: phosphorylated DARPP-32 Thr34 and Thr75
GAPDH: glyceraldehyde-3-phosphate dehydrogenase
KLH: keyhole limpet hemocyanin
RIPA: radio immunoprecipitation assay cell lysis buffer
EDTA: ethylene diamine tetra acetic acid
PFC: pre frontal cortex
ST: striatum
AMY: amygdala
HIP: hippocampus
SDS-PAGE: sodium dodecyl sulfate–polyacrylamide gel electrophoresis
BSA: bovine serum albumin
PVDF: polyvinylidene difluoride
TBS: Tris-buffered saline
TBST: Tris buffered saline containing Tween 20
S.E.M: mean ± standard error of the mean
CTR: control
UNS: unsusceptible
SUS: susceptible
PSW: passive social withdrawal
ASA: active social avoidance
