# Supplementary material for: Effects of social defeat stress on dopamine D2 receptor isoforms and proteins involved in intracellular trafficking
Source: Behav Brain Funct. 2018 Oct 8;14:16. doi: 10.1186/s12993-018-0148-5 (PMC6176509; doi:10.1186/s12993-018-0148-5)
Supplement: Supplementary file 1 — Additional file 1: Figure S1. Additional western blot results showing band of interest (D2L). Figure S2. Additional western blot results showing band of interest (D2S). [file 12993_2018_148_MOESM1_ESM.docx]

Figure S1: Additional western blot results showing band of interest (D2L).

**S1A S1B**


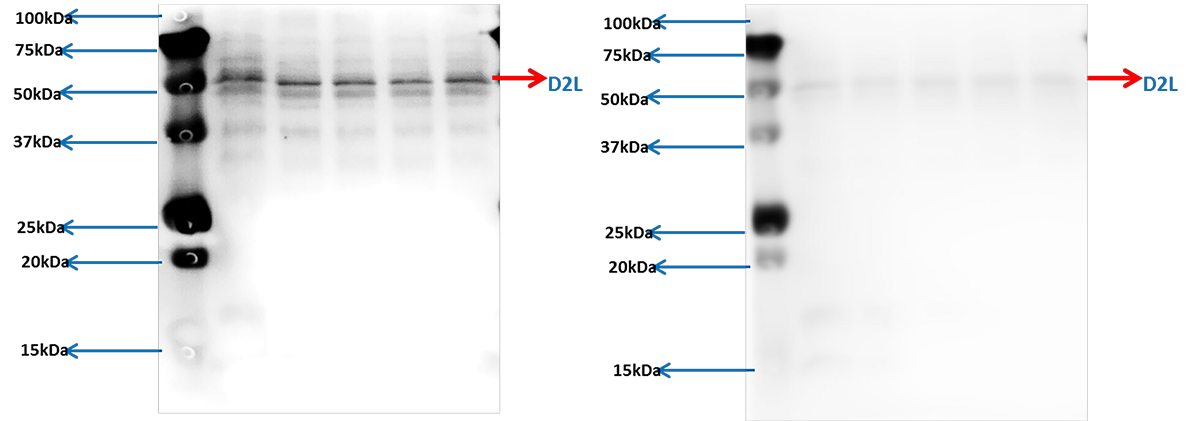


Figure S1A. Western blot results showing D2L band in whole membrane; Fig S1B. Western blot results after D2L antibody peptide blocking to confirm band of interest.

Figure S2: Additional western blot results showing band of interest (D2S).

**S2A**  S**2B**


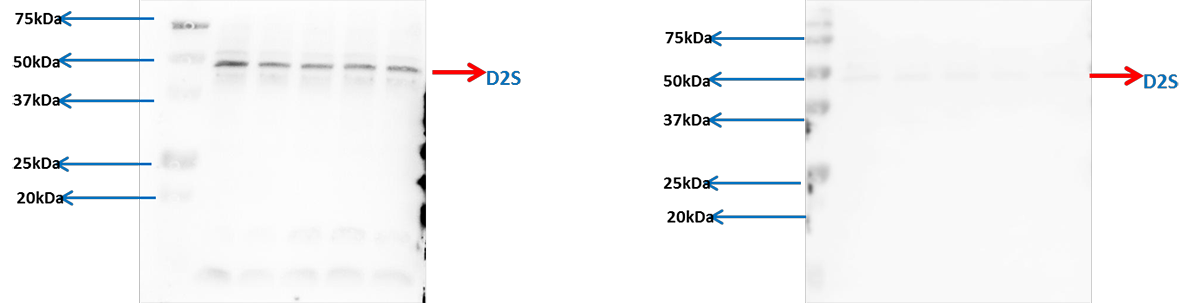


Figure S2A. Western blot results showing D2S band in whole membrane; Fig S2B. Western blot results after D2S antibody peptide blocking to confirm band of interest.
